# Symbolism of the US battlefield cross: how boots, rifles, and helmets reinforce masculinity

**DOI:** 10.3389/fsoc.2023.1148204

**Published:** 2023-05-03

**Authors:** Lauren Dundes

**Affiliations:** Department of Sociology, McDaniel College, Westminster, MD, United States

**Keywords:** battlefield cross, hypermasculinity, militarized masculinity, symbolism, boots, helmet, rifle, fallen soldiers

## Abstract

This paper explores the unconscious symbolism of the battlefield cross memorial, which is comprised of combat boots and a rifle, often with dog tags attached, topped by a helmet. While the memorial's manifest function is to provide solace, build solidarity, and convey respect for patriotic sacrifice in response to grief, the battlefield cross also exalts masculinity at a subliminal level. Because of the latent ways in which the components of the battlefield cross reinforce fallen soldiers' masculinity, the memorial provides an outlet for bereavement according to a masculine script that treats virility as sacrosanct. The resonance of the battlefield cross and its synergism with unrecognized gender coding in broader society illustrate how a powerful symbol intended to honor members of the military also valorizes machismo. This qualitative interpretation could help explain impediments to women achieving parity with men in the military.

## Introduction

The US military is a male-dominated institution with a rising number of women that comprised about 16% of the armed forces in 2020 (GAO, 2020; Robinson and Hanlon, 2020). Although many women soldiers feel supported in the military, the Department of Defense has called for renewed efforts to combat gender harassment that goes largely unreported (Department of Defense, 2020). Likewise, concerns persist about women soldiers' and veterans' feelings of discomfort, isolation, and exclusion (Rosellini et al., 2017; GAO, 2020; Reis and Menezes, 2020).^1^ In addition to formal policy remedies that aim to promote gender equality in the military^2^, scholars call for the dismantling of a “military culture of hypermasculinity” that exalts men while treating women as outsiders whose complaints justify their marginalization (Holland et al., 2016; Breslin et al., 2019; Richard and Molloy, 2020; Clary et al., 2021; Stanton, 2022, p. 7). Soldiers' internalization of idealized masculinity and anti-femininity can also engender self-doubt (and self-stigma) among those who fall short of these ideals, which in turn discourages them from seeking mental health treatment, including for military sexual trauma (MST) (Ashley et al., 2017; Andresen and Blais, 2019; Neilson et al., 2020). In addition, more subtle aspects of military culture are consistent with militarized masculinity: stereotypically masculine qualities “acquired and proven through military service or action, and combat in particular.... in which societies celebrate soldiers as ‘real men,”' to the exclusion of women and marginalized masculinities (Eichler, 2014, pp. 81–82). Despite discussion of the ontological soundness of this concept (e.g., Zalewski, 2017), militarized masculinity is useful in advancing epistemological inquiries into manifest and latent forces that hinder the recruitment and retention of women in the military as well as their higher rates of separation from the military compared to men (GAO, 2020).

**Figure 1:**
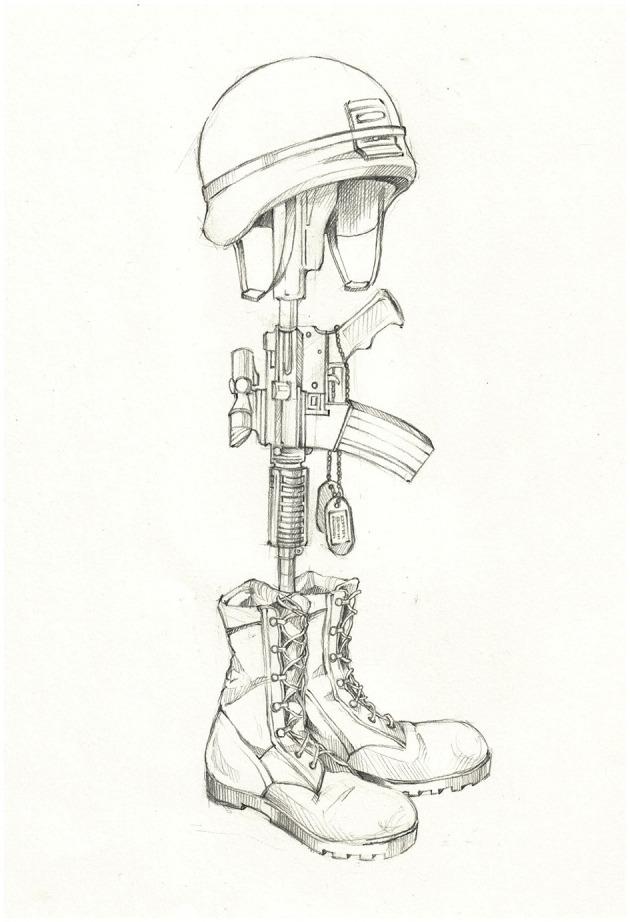
The battlefield cross.

This qualitative paper explores in depth one specific example of subliminal coding that privileges militarized masculinity: the erection of a battlefield cross (BC) to honor the sacrifice of soldiers killed in the line of duty and to mourn their loss. While its manifest function is clear—to provide comfort to the living—the BC memorial placed on the battlefield or at the soldier's base camp also exalts masculinity at an unconscious level. The BC memorial is comprised of combat boots and a downward-pointing rifle (often with dog tags attached) that is topped by a helmet (see Figure 1).

This paper presents an interpretation of the BC that suggests its symbolism reinforces masculinity post-mortem, that is, in perpetuity. Like *semper fi*, US marines' Latin motto (“always faithful”), the BC confers a sort of immortality through the consecration of these symbols as a way to resurrect the masculinity of fallen soldiers.^3^ With the understanding that the BC is polysemic, with meaning based on an individual's frame of reference, this essay parses the semiotics of the BC, especially boots, but not with the intention of foreclosing disparate or overlapping interpretations of this sacred symbol. The goal is to promote greater understanding of how underlying symbolism could contribute to resistance to military recruits, especially women, who deviate from circumscribed expectations for male gender performativity. In addition, the appeal of the BC and its synergism with unrecognized gender coding in broader society illustrate the degree to which gender hierarchy is entrenched in US culture even as the symbol simultaneously instills pride in the wake of loss.

This interpretation of how the BC helps inculcate the notion that men are best suited to be warriors is not an attempt to deny or dismiss either the practical or sentimental facets of the individual elements of the BC—nor the solace that the monument undoubtedly confers in the entirety of its assembled components. The intent is to illustrate how the BC advantages men by honoring symbols associated with masculinity. In turn, the way in which the symbolism reflects masculinity could increase the alienation that some women experience among those who grapple with “dissonance between being feminine and identifying and serving in a hypermasculine job field [that] is psychologically taxing” (Goldstein, 2019, p. 16).

## History of the battlefield cross

According to Kathleen Golden, Associate Curator in the Division of Political and Military History at the National Museum of American History, the Battlefield Cross (BC) for fallen soldiers has evolved over time:

“*Beginning with the Gulf War in 1991, and during Operations Iraqi Freedom and Enduring Freedom, the latest version of the battlefield cross, rifle, helmet, boots, and dog tags, has become the symbol of loss, of mourning and closure for the living*.

*Although it is called a cross, the memorial has no overt religious context. It's not hard to interpret the placement of the boots or the presence of the dog tags: the soldier has marched the final march to battle, and he will never be forgotten*” (Golden, 2015, para 3–4).

Because the BC is often portrayed alongside the American flag, its link to patriotism confers a pseudo-religious aura (Backhouse, 2020). While the BC may not be overtly religious, the term “cross” sanctifies the elements of the monument, endowing a type of sacrosanct status on the act of dying for one's country.

Although Golden (2015) concludes that the BC provides an eternal memorial for the soldier, its consistency across soldiers reflects group identity and not individual differences. Furthermore, “after a set period of time, the memorial is respectfully dismantled, with the components being returned to the unit for appropriate disposition” (McDonald, 2015, para 5) wherein the symbolic body of the soldier is reincorporated by the organization. By contrast, non-military graveyards tend to have a variety of gravestones with distinct epitaphs. Thus, by promoting esprit de corps over individuality, the BC serves to solidify the master status of the fallen solider as masculine hero. This elegiac recognition of masculinity in fallen warriors, in turn, consoles surviving soldiers and family members, while subconsciously exalting masculinity.

## Theoretical approach

This paper employs both social constructionism and Freudian analysis to explore the potential reasons for the resonance of the BC to memorialize fallen soldiers. In particular, elucidating the BC's phallic symbolism provides a novel perspective on militarized masculinity, beyond other analyses that recognize the overt role of war memorials in “reproducing and reinforcing masculinity” (see e.g., McDowell, 2008, p. 340). As a powerful symbol, the BC also solidifies a sense of identity among an in-group, as delineated by the social identity theory (Tajfel et al., 1979). According to this theory, individuals' group membership encourages attitudes and behaviors that reinforce the group's interests. In particular, the warrior mentality, a type of gender performativity, is spurred by contrasts with the out-group (Butler, 2006). For example, Jansen and Delahaij (2020) discuss how group members tend to protect and prioritize perceived prototypical behavior of their particular group. As a result, these authors suggest that military leadership strategy take into account how “individuals accentuate perceived similarities between themselves and other in-group members, while accentuating differences between themselves and out-group members” (p. 669). From this perspective, the BC employs symbolism, likely unconsciously, to differentiate and distinguish soldiers by “supercharging” their masculinity, even when tragedy strikes.

To more fully parse the role of symbolism, semiotic analysis is warranted. According to Peirce's sign theory, modes of thinking are grounded in context-based interpretations of signs or “representamen” (that represent or encode something else), like objects that can stand for something other than themselves. For example, objects may be perceived according to how they are interpreted as signs. Semiotics elucidates the connections between these mental representations of the external world and the internal world of ideas. Of note, Peirce believed that these ways of seeing commonly operate at an unconscious level (Danesi, 2020). Instances in which awareness of symbolic meanings could cause discomfort by challenging manifest meanings are more likely to stay invisible or unacknowledged despite their “front stage” display in what Erving Goffman called dramaturgical analysis.

How our actions resemble performers on a theater stage applies to the BC (Goffman, 1959). The BC literally is center stage in ceremonies for fallen soldiers, some incorporating it as a type of altar that affirms masculinity. This type of posthumous impression management as a front stage performance is comparable to the riderless horse in high-level memorial services (usually veterans).^4^ As the body is taken to the grave site, a riderless horse follows the casket, with a pair of empty boots in the horse's stirrups, facing backwards, as if a rider were still leading troops. In this instance, boots are a stand-in for the departed potentate, exemplifying post-mortem gender performativity. Consistent with the riderless horse ceremony, boots are perhaps the most broadly inculcated of the four symbols that comprise the BC. However, all elements of the BC coalesce to project dominance associated with masculinity. The following analysis begins at the top of the BC, with the helmet.

## Military headdresses

### Helmets

Atop the battlefield cross configuration sits the helmet, sometimes referred to as a hard hat. Helmets are obviously practical as they function to protect the head. However, the helmet-shape of the glans of the penis (Grantham, 1949; Helmet, 2006) suggests a symbolic equivalency revealed by numerous relevant slang terms for penis like purple helmet /warrior (see e.g., https://www.definition-of.com/ for terms like *helmet halva* and *helmet pelmet*). “Helmet” is also slang for condom (Appler, 2017), additional support for helmets as phallic.^5^^,^^6^ Skeptics of this assertion might consider the appearance of contemporary pith helmets of the Thailand royal guard uniform that provide a more obvious exemplification of the shared resemblance [see (Diplomat, 2020) for photo].

Helmets worn in the absence of immediate danger also serve as reminders of the perils (and presumed bravery) involved in predominantly-male professions where their use is standard. Hard hats are linked to toughness and rugged masculinity in contrast to other PPE (personal protective equipment) like respirators and hearing protection that are believed to connote weakness (Rosenberg and Levenstein, 2010). This assertion about hard hats and masculinity aligns with a reader comment on a *New York Times* article on the evolution of hard hats:

*I'm a woman who frequently visits construction sites and I'm often the only woman there. I actually feel much less conspicuous when I have my hard hat on – it looks just like everyone else's and it feels like an equalizer. However, I know a number of general contractors who keep hot pink loaner hats on site to shame workers who forget their own*. [KJ, Seattle, reader post in (Carpenter, 2019)].

Helmets share symbolism with other military hats that also have phallic qualities. For example, symbolism relevant to this paper appears in the famous late-18^th^ century print by William Heath of the Duke of Wellington, the so-called “invincible general,” recognized not only for his military prowess but also as a ladies' man. The duke's face is in profile, topped by an oversized cocked hat, also known in French as a bicorne hat for having two corners or “horns” [with horns as phallic symbols (Horn, 2018)]. The duke's hat is adorned with manifold feathers as well as tassels. Below his face is an oversized boot of his own invention (Delaforce, 2005, p. 4) still sold in his name (with the gum rubber version called wellies in the UK). His incarnation as part-boot “signaled Wellington's patriotic and masculine credentials by embodying his many martial achievements” (McCormack, 2017, p. 478) (also relevant to the discussion of boots beginning in the Boots section below). The abundant symbolism in this print at the British Museum presents Wellington as the epitome of virility, consistent with the general's famous accomplishments: “Wellington inherited Napoleon's mistress ‘*La Chanteuse de l'Empereur*”... a very satisfactory conquest” after vanquishing him at the Battle of Waterloo in 1815 (Delaforce, 2005, p. 104) (see https://www.britishmuseum.org/collection/object/P_1868-0808-8822 for this satirical print).

### Military hats and helmets with panache

The argument that helmets are phallic is consistent with historical ways in which they could be made more masculine by plumage. While feathers are polysemic [and have at times even symbolized cowardice (Gullace, 1997)], they added panache to military helmets, literally and figuratively. Panache means both “confident flair” and “a decorative plume or tuft of feathers, especially on a headdress or helmet” (Panache, 2023), evident in the feathers of war bonnets (headdresses) that symbolized male bravery in Indian nations (Koch, 1990).

To emphasize the wearer's masculinity, plumed helmets and hats were worn by high level officers, complete with tassels and an erect plume, literally embodying the notion of “a feather in one's hat.” In contrast, women's feathered hats seldom included erect feathers and were worn outside of a military milieu. Plumed bicornes followed the tradition of plumed turbans to signal bravery, modeled after the Ottoman tradition of a plume signifying military merit.

The panache dates back to cavalrymen (soldiers or warriors on horseback) beginning around 1500 and was allegedly popularized by King Henry IV of France (1553–1610) and his battle cry: “*Ralliez-vous à mon panache blanc*!”: “Follow my white plume!” (Dencher, 2015). The blatant symbolism of these helmets is highlighted in various paintings, such as an officer of France's Grenadiers of the Imperial Guard, honored by officer Napoleon François-Isidore Darquier, who wears an imposing bearskin busby hat, enhanced by both tassels and a conspicuous panache (see Thorne, 2019 for images).^7^ In addition, an exhibit *Saints and Heroes* (at the Art Institute of Chicago) displays plumage of armored men in a tournament, with a description of the exhibit noting that the actual feathers in the knights' helmets could extend up to two feet *beyond* the height portrayed in the exhibit (Antonnson, 2017).

Outside of the US, soldiers' bearskin-style busby hats sometimes have plumes (or a related form called hackles) while the elite Bersaglieri of the Italian Army currently wear feathers in their combat helmets. Their plumed helmets signal that they are an “aggressive, battle-hardened military fighting force [in which]... plumes are a badge of honor, attracting new recruits and fostering esprit and solidarity among a proud Corps of soldiers (Traficante, 2017, para 2 and 5), showing how helmets with feathers promote group unity.^8^

Likewise, both men in fiction (e.g., Robin Hood) and in reality (e.g., royalty) commonly wear one or more feathers in their headgear to signal masculinity, often with nuances conveyed by the upright positioning and feather height (see Cartwright, 2018, for striking examples among men in the British royal family). Hat-feather symbolism also exists in the unofficial anthem of the American Continental Army (in a song adopted as the state anthem of Connecticut) (Lemay, 1976): Yankee Doodle “sticks a feather in his hat” and then is exhorted to “keep it up” to be “handy” with the girls.^9^

## Guns

### Rifles

The next and center component of the BC, the rifle, evokes an armed soldier, prepared to dominate enemies. The notion of an *arm*, as in the right to bear *arms*, or in *army* or *armed* forces (with the word “force” having obvious meaning) incorporates guns (as well as other weapons, often phallic). While an arm is obviously not a phallus, it could be that an arm is upward displacement of the phallus.^10^ This assertion is supported by the meaning of a “short arm inspection,” Navy slang for when a ship's corpsman inspects a sailor's penis to check for signs of venereal disease (Navy Slang, 2021).

Beyond Freud's assertion that guns (and other weapons) are phallic (Freud, 1920), there is ample evidence of the symbolic equivalence of the penis and guns (see Trnka, 1995). Among the many examples is the risqué phrase, “Is that a gun in your pocket or are you just glad to see me?”, (falsely) attributed to film icon Mae West (Curry, 1996). Terms for male masturbation also make the connection between the penis and guns obvious:

“*Assault on a friendly weapon, buffing the rifle, cleaning your rifle, cocking the magic pistol, cocking the rifle, emptying your sex pistol, firing the love rifle, one-gun salute, shooting enemies, shooting the pump-action porridge gun, skeet shooting, target practice (with white ammo), unloading the gun*.

*Interestingly, the female counterpart, oiling the holster, places women in a receptive and secondary role”* (King, 2007, p. 90).

Other slang terms relate the penis to firearms outside of the context of self-gratification (e.g., porridge gun, love gun, yogurt rifle) (Sex-lexis, ND; Cameron, 2006; King, 2007), an association related to hypermasculinity (Neville-Shepard and Kelly, 2020). The terminology to “cock” a gun, as well as the expression to “bang” a woman (meaning sexual intercourse), also appertain. Interestingly, powder horns from animals that formerly contained gunpowder are consistent with the phallic symbolism. Horn is slang for an erection of the penis, implied in the expression “horny” and the use of the car horn to connote aggression (Horn, 2018), (another example of how sex and violence are conflated as with “bloodlust”), with relevance to shoehorns (related to the discussion of boot symbolism below).

Because guns as phallic symbols have connotations of sexual prowess and aggression, recruits must learn to focus their phallic energy appropriately (for combat). As a result, there may be a distinction between a “gun” for sexual gratification and a “rifle” as an instrument of aggression. In the words of one analyst, to transform from a civilian to a soldier, a man must “turn his affection from his girl to his rifle” (Axelrod, 2011, p. 116) to concentrate mental and physical energy on the latter. A basic internet search reveals a number of memes that are a riff on the rhyme that makes the same point: “This is my rifle, this is my gun [grabbing crotch]; This is for fighting, this is for fun [again grabbing crotch].” This verse was memorialized in popular culture following Stanley Kubrick's famous film *Full Metal Jacket* (1987) about US Marine Corps recruit training [and parodied in an episode of the animated sitcom *Family Guy* (Family Guy, 2000)].

The view of phallic energy in which sexual activity detracts from aggressive activity likely derives from the concept of “limited good” applicable to male sports (e.g., implied in the term “golf widow”) but also to battle: “A man has only so much energy and if he uses it in sexual activity, he will have that much less to use in hunting [or] warfare” (Dundes, 1978, p. 77). Despite scientific evidence that refutes this notion (Zavorsky and Newton, 2019; Zavorsky et al., 2019), the belief persists as came to light in 2018 at the Grafenwöhr, Germany-based US army unit where a detailed memo banned sexual gratification with another person (Nestel, 2019).

### Guns and politics

The phallic nature of guns is also manifested outside of the military where guns are not simply functional as a means of protection. For example, in cases of precarious manhood, stereotypically masculine behavior may help counteract negative affect and bolster manhood (Berke et al., 2017) wherein a state's level of gender role threat predicts a (compensatory) increase in firearm purchases (Cassino and Besen-Cassino, 2021). Conversely, taking away a man's gun could be the symbolic equivalent of castration, explaining why protecting the 2^nd^ Amendment appears to be more important to men than women (Lizotte, 2019).

In some instances, performative masculinity involving guns and military exploits is blatant. For example, the most prized possession of former-president George W. Bush (who served 2001–2009) was Saddam Hussein's 9-mm Glock 18C, seized when the Iraqi president was captured in Operation Red Dawn in 2003. Bush reportedly enjoyed showing Oval Office visitors the gun and telling them that it was not loaded (James, 2009). It is worth noting the following about this case in point: (1) Saddam Hussein was hunted as part of a military mission with the seized gun becoming a trophy and (2) Taking away his gun was arguably like symbolic castration. This was likely especially satisfying to George W. Bush given his father's history with the Iraqi leader that contributed to descriptions of his father (former-president George H.W. Bush) as “flaccid” and a “wimp” (Taibbi, 2018); (3) Bush emphasized that Hussein's gun was unloaded, emasculating Hussein, implying that any attempt by Hussein to defend himself would have been akin to “shooting blanks,” slang for impotence and a lack of virility.

## Boots

### Boots associated with masculine competition

Boot symbolism remains relatively unrecognized, even though the word “boots” is a metonym for soldiers (as in “boots on the ground”), which is consistent with how “the predominant symbolism of boots has been masculine and militaristic” (Steele, 1999, pp. 129) as well as more broadly symbolic of virility as conveyed by the expression “tough as old boots” (Tough as old boots, n.d.) (see Table 1). For example, a certain type of heavy, over-the-knee combat boot, the jackboot, is associated with fascism and oppressive military maneuvers, as in the expression “jack-booted thugs.” Boots represent not just power, but phallic power because feet and shoes are downward displacement of the phallus (as epitomized by the foot smashing the glass in Jewish weddings to prefigure the bride's imminent loss of virginity) (Dundes et al., 2018). Boots can also symbolize victory in football as with the Bronze Boot, the trophy in the competition between Colorado State University and the University of Wyoming in the “Border War,” which is an actual combat boot worn in Vietnam. Notably, this trophy is guarded by the ROTC unit of the most recent winner while the game is played. In another sports competition that also has battle connotations, the Holy War, the winner of the match between competing Utah schools leaves with the Beehive Boot, “the trophy of all trophies” (Niesen, 2018).^11^

**Table 1:** Ten boot expressions with gender and/or power connotations.

**Too big for your boots**	**Meaning: “cocksure.” If boot is downward phallic displacement, cockiness has outstripped the (actual) size of the boot (phallus)**
You can bet your boots	To be certain enough to risk everything (i.e., “everything” includes symbolic manhood)
Too stupid to pour piss out of a boot	If a boot is phallic, then it suggests an inability to perform bodily functions, i.e., urination
Tough as an old boot	Having great physical strength, health, or resilience
To die in (one's) boots (vs. shaking in one's boots)	Fighting back with an unwillingness to give up; to depart life with masculinity intact by displaying vim and vigor in contrast to someone “shaking in their boots” whose terror outstrips boots' phallic power
Bossyboots (describes women)	While the term bossy has been criticized for referring to strong women usurping men's authority (Black et al., 2019), bossyboots, like the term ballbuster, treats women's power as deviant^a^
To hang up one's boot	To retire from sports (with sports being a means to demonstrate virility)
Bootlicking	Willing subservience to power, without intimidation or concrete benefits (Healy, 1996; Greig, 2020)^b^
To put the boot in	To kick someone repeatedly, a reflection of the boot as a symbol of physical aggression and dominance
To get the boot, be booted out	An unambiguous dismissal or rough ejection

a Similarly, negative feedback in which men are compared to women increases men's sexism, particularly if they strongly identify as masculine (Mansell et al., 2021).

b Bootlicking is found within the BDSM community (the acronym used to describe bondage and discipline, dominance and submission, sadism and masochism) (Greig, 2020); a submissive may lick boots as a sign of deference to phallic power (Healy, 1996).

Stylistic differences in boots can heighten their phallic symbolism. Unlike basic boots in the BC, boots of higher-ranked officers may include signs of such status. For example, Hessian boots have tassels that hang down from the top center of the boot (Beard, 1999). These tassels, also found on some cocked military hats (mentioned above in the Helmets section), can connote masculinity. Like plumed helmets, tassels can connote masculinity in both hats and boots in symbolism that extends beyond the military. Turkey beards are also called tassels, with longer tassels found on mature male turkeys sought as hunting trophies (Casada, 2020; Bourjaily, 2021). In popular culture, men with long beards are often powerful, not surprising since facial hair is associated with sexual maturity (characteristic of a more fitting rival to prove masculinity).^12^

In an otherwise unexplained example, male motorcyclists, commonly concerned with machismo, may affix leather tassels (and dog whips) to their handlebars as an unconscious display of masculinity. These tassels ostensibly complement “the biker boot [that] symbolizes a big penis and an ultra-masculine persona” (Steele, 1999, p. 144). The tassel's meaning could relate back to its agricultural role in fertilization, as with the tassel on corn, the male part of the plant that produces pollen that the wind carries to fertilize the female flower (a phenomenon related to storm symbolism; see Dundes et al., 2018).

While for cowboys^13^, skinheads, and motorcyclists alike, boots serve to reinforce masculinity that characterizes a “working class (and white) view of masculinity” (Healy, 1996; Steele, 1996; Rahman and Lynes, 2018; Greig, 2020, para 10), the symbolism within a military context is especially pronounced. There are a number of expressions that show the symbolic resonance of boots, two of which directly relate to the military: (1) boots on the ground–with troops (men) reduced to boots [a term some see as dehumanizing (Wright, 2016)]; and (2) boot camp (discussed in depth below).

### Boot camps

As with the satirical print of the Duke of Wellington (discussed above in the Helmets section), in which he is reduced to a hat and boots, boot camps traditionally make men into warriors, equating them with boots. Of all the terms that could be used to identify these training camps, it is not an accident that they are called *boot* camps. Similarly, “jocks” are presumed to be male athletes who are reduced to jock straps that protect the genitals. Likewise, the word drill in “drill instructors” who are responsible for instilling military discipline, physical fitness, and weapon readiness, is likely related to its meaning to pierce and perforate (and feminize). Furthermore, “like the tool and action the word originally defines, *drill* is intended to evoke *violent and sexual* aspects of a hard-hitting lifestyle or circumstances” (Drill, 2022, emphasis added; Murphy, 2001). At a symbolic level, a sergeant who “drills” recruits treats them as subordinate, feminizing them, and modeling how performing masculinity is an antidote to this humiliation.^14^

### Boots and their power to feminize men

How boots can be instruments to feminize the enemy is apparent in Toby Keith's famous post-9-11 song *Courtesy of the Red, White, and Blue* (*The Angry American*), a tribute to military forces that aptly captured the desire to compensate for the vulnerability of the US on September 11, 2001 (a deadly attack that included the collapse of the Twin Towers of the World Trade Center and damage to the Pentagon, the headquarters of the US Department of Defense). The song was an attempt to reassert masculinity and boost morale post 9/11 by referencing patriotism.^15^

The song was a hit with US armed services in Iraq: “Bombs were branded with it. One of the first tanks into Baghdad was, as well” (Leung, 2003). The song's official video also proved popular with its show of strength featuring (mostly) men in the military, dressed in camouflage fatigues, with guns and tanks on display, and a surfeit of American flags. The most enthusiasm for Keith's song results from the line, “You'll be sorry that you messed with the U S of A... [dramatic pause].. We'll put a boot in your ass, it's the American way.” This line is followed by enthusiastic approbation of (mostly) young men in military fatigues and t-shirts printed with the word Army. In one audience-reaction scene, two young men give each other a high five, apparently at the thought of a boot in the ass of the enemy.

This imagined act of enemy humiliation through anal penetration and thus feminization is the same reasoning to “have someone's back” and to CYA (cover your ass), i.e., to prevent the dishonor of feminization through rear penetration (Dundes, 1978). The military slang IGYS “I've got your six” (referring to the position of a 6 on a clock) also conveys the same concern about feminization (Dundes, 2020). Similarly, being “under the boot” (or under the gun, for that matter) could make someone vulnerable to penetration. While this song resonated with the military (and members of the American public), the climax of the song is clearly the line about putting a boot in the rear of the aggressor, the ultimate way to shame the enemy—feminizing them, which makes sense if women are considered ill-suited to be warriors.^16^

### Boots and “phallic women”

Although feminizing women as a form of humiliation obviously has different connotations, women can appropriate the phallic connotations of boots as “phallic women” (Steele, 1999, p. 27). This exemplifies polysemy in that an object can have many different meanings, especially boots that are not combat boots, but rather are associated with feminine sexuality and power. Tall leather boots with a stiletto heel convey power in two ways: (1) Tall leather (skin-like) boots have a long shaft, making them more phallic than a short boot, with their shiny patent leather that looks wet (Steele, 1996, p. 171); and (2) The stiletto heel is also phallic, with its name—stiletto—connoting a dagger with a tapering blade. The *Kinky Boots* (2012) Broadway musical song, “Sex is in the Heel” connotes this meaning, given that the spiked heel conveys “the power to dominate and even penetrate” (Steele, 1999, p. 27). A kitten heel is a short stiletto heel, a way to feminize the phallic stiletto heel, by making it less phallic. However, although full-size stiletto boots project both the power and sexiness of a “phallic woman,” they also inhibit mobility and cater to the male gaze. This complicates the semiotics of women's heeled boots that both project the phallic power of a stiletto that can impale (as with women's acts of impalement in pegging), yet that men could also perceive as worn to please them.^17^

### The meaning of your mother wears combat boots

It is significant that the boots in the BC are combat boots that, when associated with women, convey meaning distinct from stiletto-heeled boots. The presumption of combat boots as masculine is evident in the popular insult, “Your mother wears army/combat boots.” This refrain can be understood to disparage both young men and their fathers, if the woman in the family “wears the boots” (i.e., has the phallus or balls of the family). Thus, by appropriating phallic power (boots for combat), a woman not only negates her ascribed maternal role, but also feminizes the men in the family; in other words, a woman—and not a man in the household—is the cock of the walk.^18^

It is important to point out that a similar comment about a male is far from an insult, but rather confers bragging rights, as the following internet meme reveals:


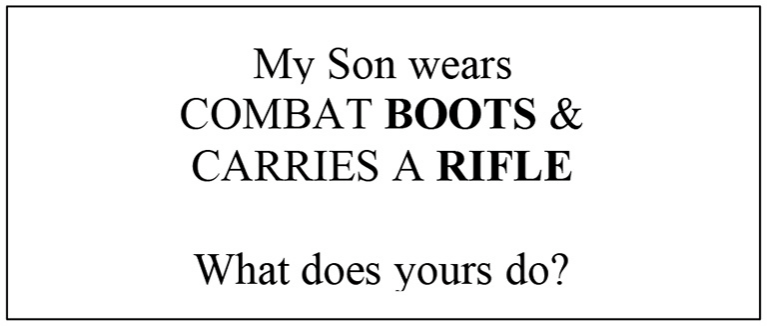


(Bolded words were presented in blue font, consistent with gendered color coding).

### Toe extensions as a measure of manhood

Historically, the horizontal length of boots could be patently phallic (which is why witches as castrating women are still commonly depicted with Crakow, extended-toe poulaine-style boots).^19^ Long-toed footwear in the Middle Ages allowed men to assert their masculinity: “Young bucks quickly exploited the phallic possibilities and soon the [shoe] extensions became longer and longer” (Rossi, 1993, p. 105). Furthermore, “medieval cordwainers stiffened the extensions to keep them erect” (Grew and de Neergaard, 2004, p. 88). Especially for men of nobility and aristocracy who were permitted longer extensions, the shoe length signaled “approaching readiness of young males to assume sexual and reproductive roles” (Rossi, 1993, p. 108). In fact, because men's shoe length continued to extend over the course of three centuries–finally reaching 24 inches longer than the foot—the shoes became difficult to walk in. This created speculation that the expression “cock of the walk” (someone whose behavior connotes presumed superiority to other group members) originated with the poulaine (Rossi, 1993); this trend perhaps also provides insight into the expression “having big shoes to fill,” which could have phallic significance regarding the ability to “measure up.” However, during the height of their popularity in England in 1463, King Edward IV denounced them as obscene, limiting their length to two inches beyond the foot, regardless of social status (Boucher, 1988).

Mexican pointy-toed boots are a modern form of poulaines: *botas picudas mexicanas* with *picuado* derived from *picar*, to prick or pierce, slang for “to sexually penetrate” in Mexico (Picar, 2022). The boot's phallic symbolism is overt: “a male peacock's mating call... for the foot” (Satenstein, 2016, para 7). All-male crews wearing pointy boots compete in dance-offs at local nightclubs playing tribal music (Simonett and Dávila, 2015), a trend that has spread beyond Mexico [for pictures see Sun (2012) and Hernandez (2015)], and that offers strong support for boots as phallic.

### Boot shining/polishing

Unlike the manifest symbolism of poulaine-style footgear, boot shining has latent meaning that has received limited attention within the military setting. In boot camp, prioritizing the care of boots is deemed to be part of general discipline, without acknowledgment of its unconscious meaning. For example, boots, as with rods or arrows (formerly called prickshafts) and other phallic symbols, have shafts (as does a penis), meaning the height of the boot rising above the foot. This not only supports the unconscious phallic meaning of a boot, but also serves as another example of how sex and aggression may conflate since the verb shaft is slang both for a man having sexual intercourse *and* treating someone harshly (Shaft, 2012), wherein both sex and violence shape conceptions of masculinity.

Boot shining is traditionally a key part of standards for the appearance of members of the military called “spit and polish.” The spit and polish standard also applies to public spheres where even “off-duty service members have a duty to project a ‘spit and polish' image to the public, wherever the public may find them” (Jurden, 2005, p. 12). In common parlance, this term denotes “extreme attention to appearance and ceremony often at the expense of operational efficiency” (Spit and polish, ND). The fact that “spit and polish” has come to mean undue or obsessive attention lends credence to special meaning of the boot.

Applying a psychoanalytic lens, boots–and guns–are polished to instill pride that also reflects their phallic symbolism. In the case of guns, sperm oil was the lubricant of choice historically (from the waxy substance found in sperm whales' head, spermaceti, that was originally wrongly identified as whale semen). Interestingly, an inferior form from bottlenose whales was sometimes incorrectly called “Arctic sperm oil.” Maintaining the shininess of jackboots, a large leather military knee-height boot, was “something of an obsession” referred to as “jacking” (McCormack, 2017, p. 472). Given the meaning of the slang term “jacking the beanstalk” as well as “jacking off,” “jacking” boots to make them shine is arguably metaphorical masturbation.^20^

This historically iconic part of boot camps, polishing or jacking boots, also may be referred to as a bullshine or spit shine (where spit was reportedly originally used as a “lubricant” to maintain high standards for extreme shininess). Bulls are symbols of masculinity (Dundes, 2020) while spitting is the symbolic equivalent of ejaculation which equates saliva to semen, explaining the term spitten (spitting) image, meaning a carbon copy (Dundes, 1991).^21^ Notably, “many examples of this expression refer to a child's resemblance to the father in particular” (Horn, 2004, p. 46). Thus, it makes sense that polishing boots unconsciously relates to masculinity, including how their shininess could signal phallic readiness.

Outside of the military, those giving and receiving shoeshines were historically male. In rare instances where women engage in the practice, the act is overtly sexual, as with the Star Shine Ladies: “youthful young women [who] polish clients' shoes in tight, revealing attire. It's like Hooters, but fewer wings” (Taylor, 2016, para 6).

### Boot polishing and hierarchy

The care of boots and shoes became linked to masculine self-sufficiency, as seen in the 14-year-old shoe shiner or bootblack, Ragged Dick, the subject of Horatio Alger's famous Bildungsroman (later serialized) about rags to respectability reflecting the Protestant work ethic. These novels strengthened the connection of boots to bootstrapping and the notion of independence implied by “pulling oneself up by one's own bootstraps” (Rooks, 2012).^22^ In addition, shoe shining as a male-only, hierarchical phenomenon involving boots helps explain how polishing someone else's boot demonstrates subservience, sometimes with sexual overtones, as in the term bootlicking (tied into bootlicking as a fetish involving dominance and submission) (Healy, 1996).^23^^,^^24^ In fact, in Supreme Court Justice Anton Scalia's scathing opposition to the admission of women to the Virginia Military Institute (VMI), he praises its Gentlemen's Code of Honor that proscribes obsequiousness characterized as “lick[ing] the boots of those above” (Justia, 1996, p. 603), a nod to the power vested in boots (even if bootlicking as a fetish was not consciously evoked).

## The symbolism of dog tags in the battlefield cross

The remaining element of the BC, dog tags, are often placed under boot laces or draped over the buttstock of the rifle. While dog tags function to provide some sense of individuality to the BC, there is also relevant symbolism related to dogs. Dog tags as a label of sorts link men to dogs, likely related to how dogs are presumed male (Leach, 2000), and thus a way to reinforce male soldiers' masculinity.

Evidence for the connection between dogs and masculinity is varied. For example, in a well-known nursery rhyme, little boys are linked to both puppies and tails: What are little boys made of? Snips, snails, and puppy-dog tails (with 2 fold symbolism given the Latin word pēnis also means “tail”). In another example, hot “dogs,” which could be any shape but look nothing like dogs, are called wieners and artificially colored to be pinkish to transform them from gray (to more closely resemble the color of Caucasian skin) (Quinlan and Watson, 2017). In addition, dogs may be called “canines” as well, a word that also means pointed teeth that are often greatly enlarged and project intimidation, a lexical meaning that complements semantic gender markers.

Various expressions also support the presumption of dogs as male, such as a man being “in the doghouse” or a salty dog, nautical slang for an experienced (male) sailor who has spent much of his life aboard a ship at sea. Furthermore, dogfights are a blood sport in which a largely male audience bets on male dogs with hopes of bolstering masculinity (Evans et al., 1998; Kalof, 2014). In contrast, in human terms, a catfight is a scuffle between women vs. a dogfight that means close combat between military aircraft. Overdogs (who are dominant) and watchdogs also carry male connotations. The alternate spelling of dog, dawg, signifies a “man, buddy, dude” while women have more associations with cats (such as cat ladies, cattiness, being subject to catcalling, and the anti-heroine Catwoman). When women act aggressively, they are said to be channeling their inner feline nature, signified by a clawing gesture accompanied by a “meow” vocalization (which implies no significant threat). On the other end of the spectrum, sex kittens are females whose display of sexuality might occur on a catwalk (vs. a runway for men), while the expression “to be weak as a kitten/cat” conveys vulnerability, related to being a scaredy-cat.

In Toby Keith's song (mentioned above in the Boots section), the stanza preceding the “boot in your ass” line likens the US military to a “big dog” that “will fight when you rattle his cage” with martial repercussions for those that “messed with the USA”, reminiscent of the World War I battle of Belleau Woods that spawned the Marines' name “Devil Dogs,” a prized moniker signifying ferocity in combat.

An additional means of exploring the gendered implications of a dog tag is through analysis of the word bitch. A woman deemed “dominant” might be called a “bitch” (Kleinman et al., 2009, p. 47). This could be because if dogs are seen as males and males are supposed to be dominant, an overbearing woman viewed as upending male power would reverse the presumed male gender of dogs (and their top dog status). Likewise, a male who is subservient to a female is still associated with being a dog (as a male), but if he is not the alpha, it is emasculating and castrating, reassigning him as a female dog (bitch).

Thus, a bitch is either a strong woman (counter to her ascribed role) or a weak male (also counter to his ascribed role), semantic meaning embedded within the anthropomorphic semiotics of dogs presumed to be male. This also helps to explain why women who call each other bitch may find it empowering (an unconscious recognition of upending male-based power) while men are unlikely to feel empowered by calling each other bitches or pussies (who pussy*foot* around and beat around “the bush”), terms that imply weakness.

An interesting exception is the fabled (male) swashbuckler, Puss in Boots. Although a “puss” (a cat) is not inherently masculine, a puss *in boots* has different connotations. That is, when boots are incorporated into the name of the heroic feline, boots' masculine associations telegraph his virility. Furthermore, Puss in Boots also wields a long sword and wears a tricorn hat with a long feather, confirmation of his machismo.

## Practical implications

The aforementioned gender-related associations of the elements of the BC imply that masculinity is encoded in a way that complements the BC's overt function of remembering a fallen soldier—especially apropos for soldiers that are men. While the symbolism of the BC conveys appreciation for military sacrifice by emphasizing masculinity, particularly phallic masculinity, women may be less apt to be perceived as consummate soldiers. The possibility of these perceptions persists in contradistinction to Scythian culture, where women referred to as Amazons had parity with men in battle. Analysis of grave-site artifacts and remains found in Scythian *kurgans* (grave mounds) in the southern Ural Steppes near the Kazakhstan border in Russia have corroborated artistic renderings of such equal participation, e.g., battle wounds (arrowheads embedded in bones) and the burial of women with adjacent weapons (Mayor, 2016).

Reports that these women warriors were fictional diminishes their actual participation in battle as archers on horseback, despite evidence from the remains of a woman whose bowlegged condition resulted from extensive riding (Mayor, 2014) (gender parity reminiscent of men and women competing equally, without gender divisions, in Olympic horseback riding). However, treating only men as warriors and excluding or minimizing the role of women in these martial roles increases dependence on men and reinforces their dominant position in society (Mayor, 2014), a portrayal that helps maintain the primacy of masculinity that extends postmortem in the case of the BC.

## Conclusion

At the core of the BC is its glorification of masculinity, with latent symbolic meaning attached to each of its components. The BC both reflects and enshrines virility, even as we must recognize that this memorial does not capture the complexities and fluidity of gender scripts relevant to a soldier's death. For example, grieving mothers who are “devoid of symbolic and concrete power” support a hegemonic model of bereavement. In their apolitical memorialization and veneration of their sons as patriots, “they exchange their trauma for moral and symbolic capital and penetrate the public discourse by carrying the flag of their loss and bereavement [by] acting within the confines of a gendered worldview” (Lebel and Hermoni, 2018, p. 136 and p. 145).

Other important considerations in recent military-related discourse involve the changing nature of combat and the evolving role of women in warfare that undercut the heroic solider myth. In particular, the growing use of drones in warfare raises questions about whether combat confers or upends militarized virility (Millar and Tidy, 2017). Because honor and courage have been associated with the risk of self-harm, drone pilots that employ finesse and mental toughness from a safe, remote office are inadvertently challenging the traditional emphasis on physical strength that has been central to militarized masculinity (de Volo, 2016). Furthermore, drone pilots also may be part of covert operations that can bring accusations of cowardice (de Volo, 2016). Furthermore, the masculinity of both those responsible for remote-controlled strikes as well as the male targets of those drone strikes could be considered superfluous (de Volo, 2016).

Despite the evolution of militarized masculinity, the BC continues to exemplify how a surfeit of powerful symbolism feeds into the notion that male bonding is fundamental to military prowess, camaraderie that confers masculinity reinforced by largely invisible social cues in broader society. The BC, a memorial that exudes symbolic masculinity, is subsumed into the warrior culture paradigm that is credited with making the US a strong country, impenetrable to enemies. As a traditionally homosocial institution, the military continues to promulgate the unambiguous masculinity of soldiers and downplay or ignore the contributions of female soldiers (McDowell, 2008; Pendlebury, 2020; Szitanyi, 2020). This increases pressure on women in the military to assimilate into the male-dominated culture in order to be accepted as “one of the guys” (Meade, 2020, p. 51). These phenomena contribute to the intransigence of militarized masculinity, despite countermeasures such as the Department of Veterans Affairs' revised mission statement in which soldiers are no longer presumed to be men (Kime, 2023).

The values represented in the BC promote a line of thinking that functions as a recruiting technique that (consciously or not) perpetuates hypermasculinity while reinforcing conventional gender roles. The means for soldiers to gain the imprimatur of masculinity also raises questions about women's fitness for active military service:

“[A military recruit is] probably more than ready to accept the military's gift to him of the image of enhanced [phallic] power. The military, in turn, is more than happy to give its recruits an image of sexual power if they are willing to fight and die for it” (Trnka, 1995, p. 239).

Along the same lines, the phrase “wounded warrior” compensates for wounded men's inability to fight by attaching the tag of warrior (i.e., once a warrior, always a warrior, in accordance with the social identity theory). By conflating hypermasculinity with patriotism and valor, cultural cues like those in the BC function to encourage military service despite the horrors of war.

Symbolism of the BC also suggests that having women in the military conflicts with the BC's embodiment of masculinity, a nexus that could deter women but that also could attract voluntary warriors seeking not only to “serve their country” but also an imprimatur of masculinity. Performing masculinity does not end with a soldier's death, but rather is re-broadcast by the BC on a frontstage platform. From this perspective, erecting BCs could help assuage doubts that might surface during the course of this perilous commitment. At the same time, the symbolic phallic resonance of the BC also could play a role in solidifying the maleness of the military while minimizing women's comfort and influence in military matters. To expedite substantial progress toward gender parity, we should take into account how symbolism links masculinity to military prowess.

## Data availability statement

The original contributions presented in the study are included in the article/supplementary material. Further inquiries can be directed to the corresponding author.

## Author contributions

The author confirms being the sole contributor of this work and has approved it for publication.

## Footnotes

^1^Some women soldiers have described how their gender became their master status as their femininity was ridiculed, with gender-based hazing exacerbated by ill-fitting unisex uniforms designed for men (Goldstein, 2019). They are also subject to resentment over gender-modified “adversative” techniques (e.g., taxing physical activity, mental strain, and exacting behavior directives) traditionally used to instill military discipline (Do and Samuels, 2021; Schaefer et al., 2021).

^2^Recognition of gender bias in the military led to the Deborah Sampson Act (2021) to better serve women veterans, including survivors of military sexual trauma (MST). The act is named in honor of one of the first American women to serve with distinction in combat (in 1782-3) who also felt compelled to disguise herself as a man (Stanton, 2022).

^3^Uses of the BC to honor fallen soldiers ranges from tattoos to farewell rites. See, for example, news coverage of the ceremony at Bagram Airfield in Afghanistan that commemorated US airmen who perished when their C-130J Super Hercules crashed shortly after takeoff from Fratini (2016).

^4^In, 2004, an Old Guard riderless horse-drawn caisson carried the coffin of President Ronald Reagan to the Capitol, a rite accorded also to Herbert Hoover, Lyndon B. Johnson, General Douglas MacArthur, and President John F. Kennedy (Faulkner, 2008).

^5^The phallic connotations of helmets are overt in Trojan brand condoms, with their helmet insignia, now synonymous with condoms. Similarly, Star Wars brand condoms show a helmeted stormtrooper with the caption: Always wear a helmet (RipnRoll, 2021). Likewise, DKT's Kiss brand condoms have a tagline warning that wearing a covid facemask below the nose is like riding without a helmet (with a helmet pictured as a condom), consistent with the words hats and caps that also are condom argot (Amy and Thiery, 2015).

^6^Beyond the military, helmets can connote virility. E.g., in groundbreaking ceremonies, men traditionally wear “hard” hats or helmets to penetrate the earth using a commemorative shovel to celebrate the impending *erection* of an edifice. This same ritual is also called a sod turning ceremony, a reference to loosening soil prior to planting seeds, like the functions of *plowing and sowing*, Biblical metaphors for the man's role in procreation (James, 2017). Akin to the US concept of mother earth, “earth is always conceived as female in Mesopotamian myths of the creation genre” as in the Babylonian creation story, the Harab “Plow” myth,” when conception occurs with the “stroke” of a plow (Lambert, 2013, pp. 387, 393). This perhaps suggests that ribbon cutting ceremonies after the erection of a building symbolize cutting the umbilical cord (and male usurpation of women's role in creation).

^7^The winkie soldiers in the *Wizard of Oz* (1939) wear plumed busby hats, carry pikes, and chant in deep voices, masculinity that is undermined when they are under the control of the Wicked Witch of the West until she is destroyed and Dorothy is able to take her broomstick to the impuissant Wizard who presumably wants to re-appropriate the phallic power the broomstick confers.

^8^Football-helmet-display-case cabinets and home football flags commonly feature only a helmet and team name that shows a certain reverence for helmets, reflecting the sport's symbolism related to male dominance (Dundes, 1978). Its players risk serious injury in a sport with no female-team counterpart.

^9^In Disney's *Dumbo* (1941), the eponymous elephant hero feels empowered to fly because of a feather he carries upright in his trunk. Dumbo wears a phallic cap while his mentor mouse has an erect feather in his cap. After Dumbo proves himself, the mouse signs a contract for Dumbo with an oversized feather quill pen, another symbol of masculinity (see Dundes et al., 2018) for the phallic power of pens and signing.

^10^Similarly, maidenhead is upward displacement meaning virginity, and not literally a maiden's head.

^11^This theme is reminiscent of drinking competitions involving a glass shaped like a boot that is marketed as an ideal gift for groomsmen (see e.g., *bierstiefel* on Amazon.com).

^12^In the classic fable of the “Three Little Pigs,” the male pigs' bravado is expressed in their response to the big, bad wolf who bellows, “Open the door and let me in!”. In answering, “Not by the hairs on my chin-y chin chin” they allude to their non-existent beards, perhaps explaining why the house made of bricks, and not their machismo, saves them.

^13^Cowboy boots were modeled after the military footwear of Hessian soldiers who fought in the American Revolutionary War (Beard, 1999), with a sexually suggestive rising toe cap.

^14^In the film *GI Jane* (1997), the special operations recruit, portrayed by Demi Moore, finally wins her peers' respect when she “performs masculinity” by enduring a beating by the Master Chief, “taking it like a man.” The scene culminates in her shouting, “Suck my dick,” to the cheers of her (male) peers. This supports the notion that women in the military must appropriate and embrace phallic power—as a so-called “phallic woman.”

^15^Patriotism has the same masculine root as for the word father, *patriota* “fellow countryman” in Latin, and *patrios* “of one's fathers”, from *patris* “fatherland” from Greek (Patriotism, 2011). Not coincidentally, the surface-to-air Patriot missile system reflects the fusion of fatherhood, patriotism, and weapons.

^16^This also explains why “kicking someone's ass” may have nothing to do with a person's rear end, but rather signifies a target's clear and humiliating defeat that symbolically involves a foot and the target's derrière.

^17^Ambivalence over integration of women into the military and gender equality surfaced in the form of controversy about appropriate shoes for women in the military in 2021 in Ukraine, when women marched in a parade in heels, a sign of their “incapability,” which created controversy and backlash that garnered attention internationally (Peltier and Varenikova, 2021).

^18^Some women in the military, particularly lesbians, earn the status of “*honorary males*” (Carreiras, 2008; Ely and Meyerson, 2010). This contrasts with the rejection of gay men whose identities may be “conflated with femininity” (Van Gilder, 2019, p. 11). This thinking applies in Brazil, where footwear symbolism is relevant: *sapatao*, literally “big shoe,” is slang for lesbian while the term *coturno* “army boot” emphasizes a woman's manly qualities. In contrast, a “femme” lesbian is called a *sapatilha* (a slipper worn by ballerinas) (Steele, 1996, pp. 104–5).

^19^A witch costume calls not just for pointy shoes or boots, but also a pointy hat, complemented by a long pointy nose and chin, pointy nails (like talons), and pointy teeth—all of which could be seen as projecting an appropriated phallic penetrative threat.

^20^This slang term is consistent with the phallic nature of the beanstalk in the tale in which Jack defeats the giant—a father substitute—by chopping down the beanstalk (Hardison, 2017). This Oedipal act of symbolic castration results in the giant's death, allowing Jack and his mother to live happily ever after. In popular culture, the third season of the show *Succession* (2021) portrays a father-son rivalry or so-called “Oedipussy” theme in which the patriarch opens the season with the words, “It's war”. He later parrots the threat of the fabled giant: “I'll grind your bones to make my bread” to which the son retorts, “Well, then... I'm gonna run up the f^***^in' beanstalk” (Snow, 2018).

^21^In gender reveal parties, the hosts pop a confetti-filled balloon or *cannon* popper or set off a glitter *bomb* to reveal gender-stereotyped pink or blue coloration (reinforcing the gender binary). The possible underlying meaning of the pseudo-ejaculation has parallels with the man determining the baby's sex via his sperm, the front end of the cradle-to-grave celebration of phallic power.

^22^Horatio Alger has been exposed as a pedophile (Polenberg, 1997; Bateman, 2004).

^23^Boot fetishes are relevant to leather events catering to gay men. At these parties, a bootblack may be available to shine the boots of attendees who sit in an elevated chair resembling the set-up of a traditional shoeshine stand. The bootblack is generally also available to perform other services for customers. Such events may be concurrent with the International Mr. Bootblack competition (IMrBB), part of the International Mr. Leather conference that celebrates hypermasculinity in the gay community. The phallic symbolism of boots at this event also applies to role playing interactions in which boots are complemented by a full military uniform to signify dominance (Healy, 1996).

^24^Because the military emphasizes strict deference to authority within a pecking order (where “peckers” determine one's social position), military attire appeals to the BDSM community given “how fetishism and militarization concatenate” and how “male dominants are almost always dressed in quasi-military uniforms” (Crane-Seeber, 2016, pp. 3, 9). In the military, “rituals of rank, uniforms, regimented life, privileged sexual and social status, a feeling of superiority to civilians [are] analogous to a quasi-consensual 24/7 BDSM relationship with the state” since “status in the military is earned for being compliant, skilled, and pro-actively obedient” (Crane-Seeber, 2016, pp. 5, 10).
